# Hypertension Is Associated with Marked Alterations in Sphingolipid Biology: A Potential Role for Ceramide

**DOI:** 10.1371/journal.pone.0021817

**Published:** 2011-07-19

**Authors:** Léon J. A. Spijkers, Rob F. P. van den Akker, Ben J. A. Janssen, Jacques J. Debets, Jo G. R. De Mey, Erik S. G. Stroes, Bert-Jan H. van den Born, Dayanjan S. Wijesinghe, Charles E. Chalfant, Luke MacAleese, Gert B. Eijkel, Ron M. A. Heeren, Astrid E. Alewijnse, Stephan L. M. Peters

**Affiliations:** 1 Department of Pharmacology and Pharmacotherapy, Academic Medical Center, Amsterdam, The Netherlands; 2 Vascular Medicine, Academic Medical Center, Amsterdam, The Netherlands; 3 Department of Pharmacology and Toxicology, Maastricht University, Maastricht, The Netherlands; 4 Department of Biochemistry, Virginia Commonwealth University, Richmond, Virginia, United States of America; 5 FOM Institute for Atomic and Molecular Physics, Amsterdam, The Netherlands; City of Hope National Medical Center and Beckman Research Institute, United States of America

## Abstract

**Background:**

Hypertension is, amongst others, characterized by endothelial dysfunction and vascular remodeling. As sphingolipids have been implicated in both the regulation of vascular contractility and growth, we investigated whether sphingolipid biology is altered in hypertension and whether this is reflected in altered vascular function.

**Methods and Findings:**

In isolated carotid arteries from spontaneously hypertensive rats (SHR) and normotensive Wistar-Kyoto (WKY) rats, shifting the ceramide/S1P ratio towards ceramide dominance by administration of a sphingosine kinase inhibitor (dimethylsphingosine) or exogenous application of sphingomyelinase, induced marked endothelium-dependent contractions in SHR vessels (DMS: 1.4±0.4 and SMase: 2.1±0.1 mN/mm; n = 10), that were virtually absent in WKY vessels (DMS: 0.0±0.0 and SMase: 0.6±0.1 mN/mm; n = 9, p<0.05). Imaging mass spectrometry and immunohistochemistry indicated that these contractions were most likely mediated by ceramide and dependent on iPLA_2_, cyclooxygenase-1 and thromboxane synthase. Expression levels of these enzymes were higher in SHR vessels. In concurrence, infusion of dimethylsphingosine caused a marked rise in blood pressure in anesthetized SHR (42±4%; n = 7), but not in WKY (−12±10%; n = 6). Lipidomics analysis by mass spectrometry, revealed elevated levels of ceramide in arterial tissue of SHR compared to WKY (691±42 vs. 419±27 pmol, n = 3–5 respectively, p<0.05). These pronounced alterations in SHR sphingolipid biology are also reflected in increased plasma ceramide levels (513±19 pmol WKY vs. 645±25 pmol SHR, n = 6–12, p<0.05). Interestingly, we observed similar increases in ceramide levels (correlating with hypertension grade) in plasma from humans with essential hypertension (185±8 pmol vs. 252±23 pmol; n = 18 normotensive vs. n = 19 hypertensive patients, p<0.05).

**Conclusions:**

Hypertension is associated with marked alterations in vascular sphingolipid biology such as elevated ceramide levels and signaling, that contribute to increased vascular tone.

## Introduction

Hypertension is a major risk factor for cardiac, cerebrovascular and renal disease. It is associated with increased vasomotor tone, decreased vasodilator potential and inward remodeling of blood vessels. The presence of vasomotor imbalance in essential hypertension is partly mediated by decreased nitric oxide bioavailability and elevated release of endothelium-derived contractile factor (EDCF) as characteristics of endothelial dysfunction, and impaired smooth muscle cell responsiveness towards relaxing factors [1], [2]. Regulation of vascular reactivity and cellular growth have been shown to be partially mediated by an intrinsic network of bioactive lipids classified as sphingolipids, of which sphingomyelin is abundantly present in virtually all cells.

Sphingomyelin is an ubiquitous membrane (sphingo)phospholipid that may serve as a substrate for sphingomyelinases for the production of ceramide [3]. Ceramide can be further converted into ceramide-1-phosphate (C1P), glucosylceramide or sphingosine by phosphorylation, glucosylation or deacylation, respectively. Subsequently, sphingosine can be phosphorylated by sphingosine kinases to yield sphingosine-1-phosphate (S1P), which can target five G-protein coupled S1P receptors (S1P_1–5_), of which S1P_1–3_ are expressed in the cardiovascular system [4]. S1P receptor activation induces proliferation of many cell types including vascular cells [5]. Conversely, sphingosine and ceramide, the precursors of S1P, have growth-inhibiting and pro-apoptotic actions [6]. Because of these opposing actions of sphingomyelin metabolites, this system is also referred to as the ceramide/S1P rheostat [7]. In addition to these growth-regulating properties, we and others have shown that sphingolipids are involved in the regulation of vascular tone, for instance by regulating nitric oxide and EDHF-mediated relaxing responses in different types of blood vessels [8]–[10].

Because sphingolipids are involved in the regulation of both vascular growth and vascular tone, we hypothesized that in essential hypertension, sphingolipid ratios are altered, resulting in an altered vasomotor function. Here we show that 1) Elevation of vascular ceramide leads to vasoconstriction due to increased TXA_2_ release in vessels of SHR. 2) These constrictions are only observed in vessels of SHR due to increased expression of enzymes involved in thromboxane A_2_ synthesis. 3) That basal ceramide levels are elevated in both SHR and humans with hypertension.

## Results

### Modulation of sphingolipid metabolism induces transient constrictions in isolated SHR carotid artery

**Figure 1:**
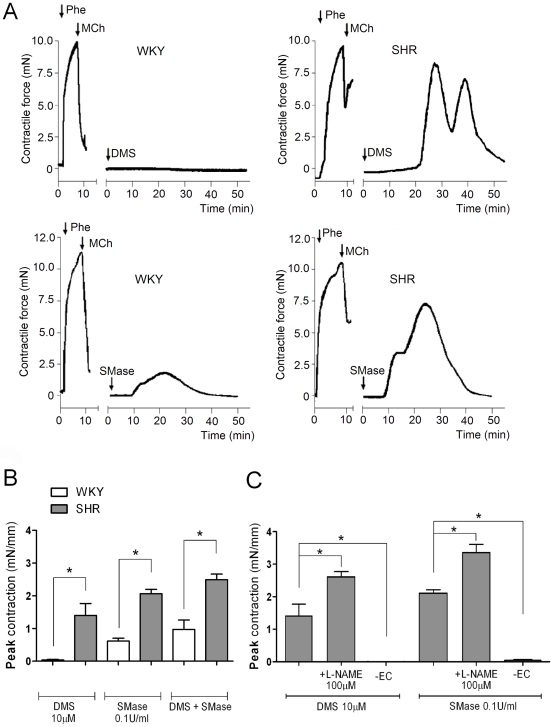
DMS-induced and SMase-induced contraction in SHR and WKY carotid artery. A) Original tracing of rat carotid artery segments exposed to DMS (10 µmol/L) or SMase (0.1 U/mL). Note reduced relaxing response to MCh (10 µmol/L) in SHR, indicating pronounced endothelial dysfunction. B) Maximal contractile responses to DMS and/or SMase in intact WKY and SHR vessels and C) SHR vessel responses in the presence of L-NAME or endothelium-denudation (-EC). Phenylephrine (Phe), methacholine (MCh), dimethylsphingosine (DMS), sphingomyelinase C (SMase), N^ω^-Nitro-L-arginine methyl ester (L-NAME). Data presented as mean ± SEM, n = 5–6, (*) p<0.05.

Contractile responses of isolated carotid artery segments to K^+^ (100 mmol/L) and phenylephrine (Phe; 0.3 µmol/L) were slightly reduced in vessels of SHR compared to WKY (Table 1). Endothelium-dependent relaxation to methacholine (MCh; 10 µmol/L) during Phe pre-contraction was impaired in SHR, reflecting endothelial dysfunction (maximal relaxation: 91±1% WKY vs 50±1% SHR, n = 10, p<0.05, Fig. 1A and Table 1). Incubation of the carotid artery segments with the sphingosine kinase inhibitor dimethylsphingosine (DMS; 10 µmol/L) or dihydrosphingosine (DHS; 30 µmol/L, data not shown) induced a marked transient contraction in SHR vessels, which was absent in age-matched WKY rats (Fig. 1A). In addition, exogenously applied neutral sphingomyelinase (SMase; 0.1 U/mL) evoked similar contractile responses in SHR vessels that were much less pronounced in vessels of WKY (Fig. 1A). When DMS and SMase were applied simultaneously, contraction was slightly higher, but not synergistically elevated (Fig. 1B), suggesting a similar mechanism of action. Importantly, contractions induced by DMS or SMase in the SHR carotid artery were completely abolished by mechanical removal of the endothelium. In contrast, the nitric oxide synthase inhibitor L-NAME significantly increased DMS-induced and SMase-induced contractions (Fig. 1C).

**Table 1:** General characteristics of anaesthetized SHR and WKY rats and *ex vivo* carotid artery segments.

Parameters	WKY	SHR
n	10	10
Weight, gram	482±8	360±6 *
MAP, mmHg (under isoflurane)	71±3	95±6 *
Blood flow, mL/min (systolic, carotid a.)	28±3	18±2 *
Heart rate, BPM (under isoflurane)	343±10	309±5 *
Lumen Ø, µm (segment at 90 mmHg)	1119±122	1069±9 *
Constriction, mN/mm (3^rd^ K^+^)	4.1±0.2	3.5±0.1 *
Relaxation, %Phe preconstriction	91±1	50±1 *

Data expressed as mean ±SEM, (*) p<0.05.

### Sphingomyelinase-induced contractions require cyclooxygenase-1 and involve elevated thromboxane A_2_ production

**Figure 2:**
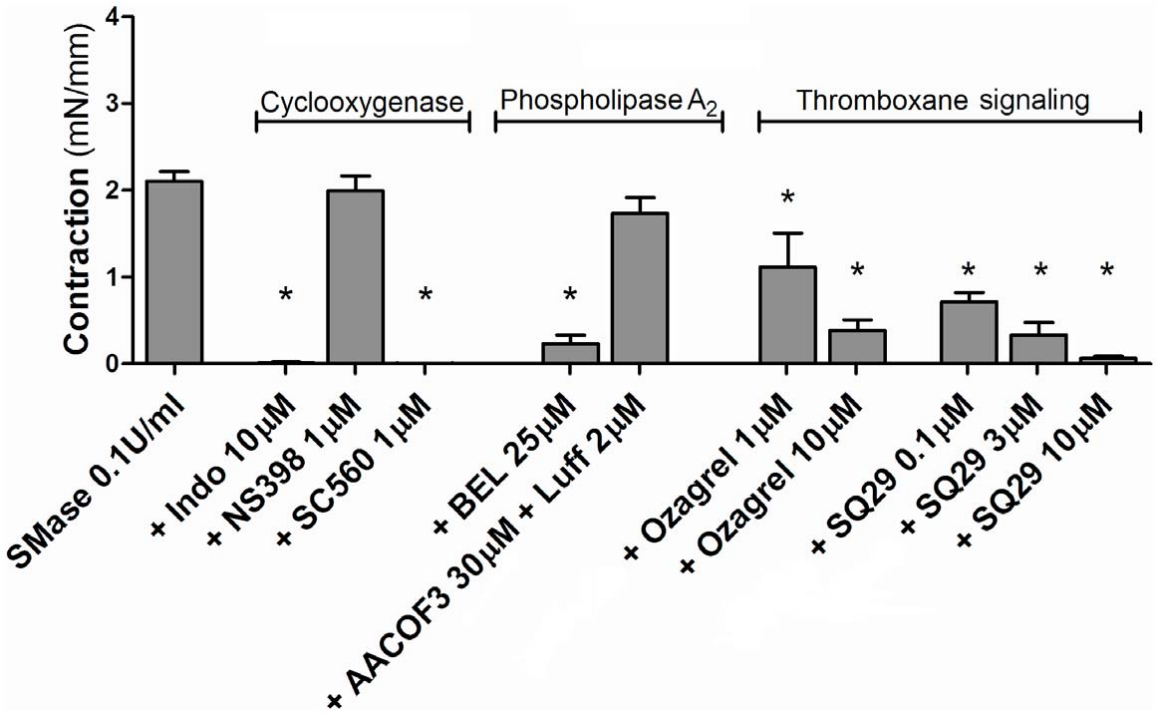
Characterization of SMase-induced contraction in SHR carotid artery. SMase-induced contraction, in absence and presence of the non-specific COX inhibitor indomethacin (Indo), COX-1 specific inhibitor SC560, COX-2 specific inhibitor NS398, the PLA_2_ inhibitors AACOF3 (cPLA_2_) and Luffariellolide (Luff; sPLA_2_), Bromoenol lactone (BEL; iPLA_2_), the thromboxane synthase inhibitor Ozagrel and the thromboxane receptor antagonist SQ29548 (SQ29). Data presented as mean ± SEM, n = 4–6, (*) p<0.05 compared to control SMase.

**Figure 3:**
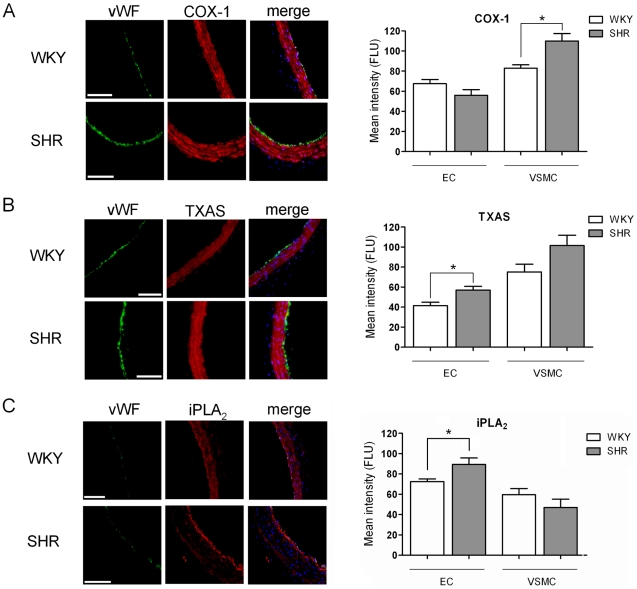
Immunohistochemistry of relevant proteins in SHR carotid artery. A) Immunohistochemical staining (left, typical staining images; 200× magnification) and quantification (right) of SHR or WKY carotid artery segments depicting cell nuclei staining (blue), with/without the von Willebrand Factor (vWF) endothelium marker (green) and cyclooxygenase-1 (COX-1; red). B) thromboxane synthase (TXAS; red) and C) calcium-independent phospholipase A_2_ (iPLA_2_; red). Please note the increased EC/VSMC iPLA_2_ expression ratio in SHR. Data presented as mean ± SEM, n = 5–6, (*) p<0.05, scale bars 100 µm.

The non-selective cyclooxygenase (COX) inhibitor indomethacin (10 µmol/L) and the COX-1 selective inhibitor SC560 (1 µmol/L) entirely prevented SMase-induced contraction in SHR carotid artery (Fig. 2), while the COX-2 selective inhibitor NS398 (1 µmol/L) was without effect. Applying higher concentrations of NS398 resulted in decrease contractile responses, however, at these concentrations NS398 is reported to non-specifically inhibit COX [11]. Since COX products include contractile prostaglandins and thromboxane, we applied the thromboxane/prostaglandin (TP) receptor antagonist SQ29548, which concentration-dependently inhibited SMase-induced contractions. Furthermore, the thromboxane synthase (TXAS) inhibitor Ozagrel concentration-dependently inhibited vascular contraction (Fig. 2). In order to investigate whether the sensitivity of agonist-induced TP receptor activation was different for SHR and WKY carotid arteries, we generated concentration-response curves for the thromboxane analogue U46619, which was not different between SHR and WKY (Fig. S1). Immunohistochemical quantification of COX-1 and TXAS expression showed that COX-1 was elevated in SHR smooth muscle cells compared to WKY (Fig. 3A). TXAS expression in SHR was significantly elevated compared to WKY in endothelium (Fig. 3B).

### Calcium-independent phospholipase A_2_ is the source of arachidonic acid in SMase-induced contractions

To investigate which enzyme was mainly responsible for generating the COX substrate arachidonic acid, several phospholipase A_2_ (PLA_2_) inhibitors were applied. The individual or combined addition of inhibitors of cytosolic PLA_2_ (AACOF_3_; 30 µmol/L) or secretory PLA_2_ (Luffariellolide; 2 µmol/L) to SMase-induced contractions were without effect. However, the calcium-independent PLA_2_ (iPLA_2_)-specific inhibitor Bromoenol lactone (BEL, 25 µmol/L) significantly inhibited SMase-induced contractions (Fig. 2). In line with this, immunohistochemical quantification indicated increased expression of iPLA_2_ in the endothelium and a decreased expression in smooth muscle cells of SHR. Accordingly, the ratio of EC/VSMC iPLA_2_ expression was markedly shifted towards the endothelium in SHR compared to WKY vessels (Fig. 3C). Imaging mass spectrometry and experiments with SMaseD (which generates C1P directly from sphingomyelin) revealed that ceramide (and not C1P) is most likely responsible for iPLA_2_ activation (Fig. S2, Fig. S3).

### In vivo administration of DMS results in a marked rise of blood pressure in SHR but not WKY

**Figure 4:**
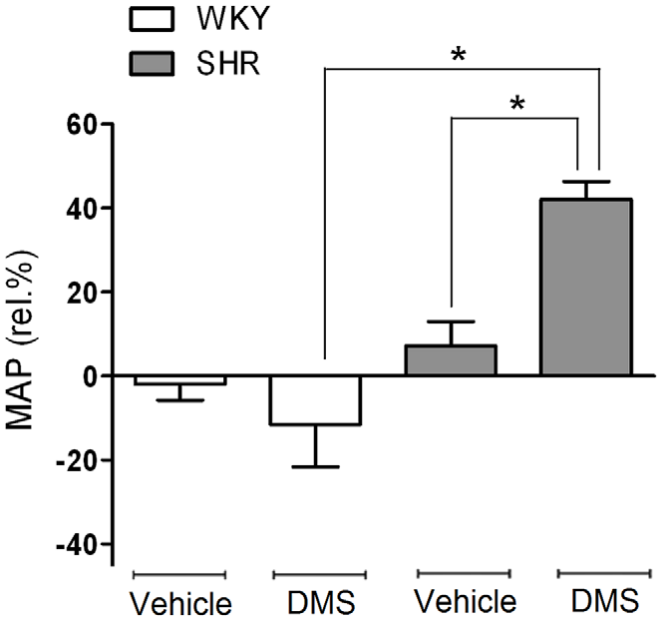
*In vivo* effects of DMS infusion in SHR and WKY. Rats were treated with bolus injection and subsequent infusion of DMS (3 mg/kg followed by 6 mg/kg/hr) or vehicle (0.75% rat serum albumin in saline) during recording of mean arterial pressure (MAP). Data expressed as mean maximal change from baseline ± SEM, n = 6–8, (*) p<0.05.

To investigate whether sphingolipid modulation also differentially affects blood pressure *in vivo*, DMS was applied i.v. to isoflurane-anesthetized SHR and WKY. Arterial blood pressure was measured, as well as common carotid blood flow using a transit time flow probe. Baseline hemodynamic values that were obtained after stabilization of the preparation are shown in Table 1. Mean arterial pressure was substantially higher in SHR than in WKY. In SHR, application of DMS, but not vehicle, resulted in a significant increase in mean arterial pressure (Fig. 4), accompanied by a further rise in carotid artery resistance and slightly decreased heart rate (Fig. S4). In WKY however, DMS had little effect on blood pressure. The heart rate was not significantly different between SHR and WKY after DMS administration.

### Ceramide levels are increased in arterial tissue of SHR

**Figure 5:**
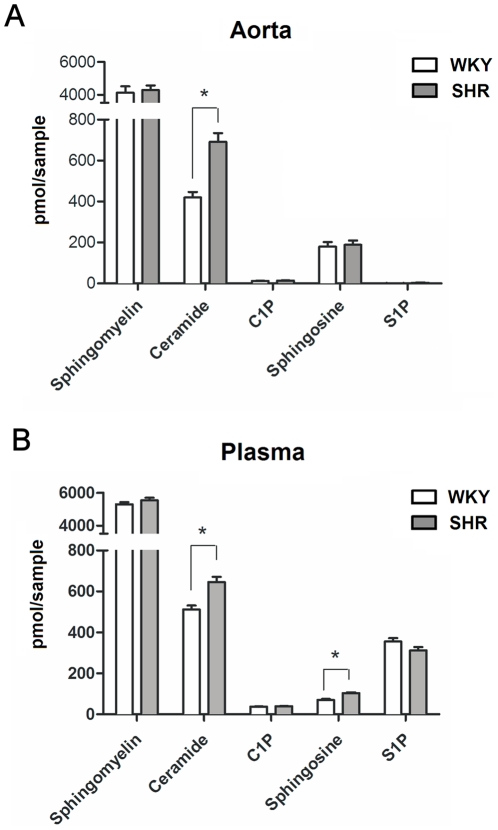
Liquid-chromatography-mass spectrometry measurements of total sphingolipid content. Content was measured in A) SHR and WKY aorta homogenate and B) blood plasma. Data presented as mean ± SEM, n = 3–5 (for 6–10 pooled aortas) and 6–12 (for plasma samples), (*) p<0.05.

Mass spectrometric analysis [12] revealed significantly increased levels of total ceramide in arterial tissue (aorta) of hypertensive rats compared to normotensive rats. No significant changes in total sphingomyelin, C1P, sphingosine and S1P were observed (Fig. 5A). The significant increase in total ceramide was mainly due to increased C16:0, C18:0 and C24:1 ceramides (Fig. S5).

### Plasma ceramide levels are increased in both hypertensive rats and humans

Mass spectrometric lipidomics analysis revealed increased total ceramide levels and slightly increased sphingosine levels in blood plasma from SHR (Fig. 5B). The increased total ceramide levels were mainly due to increases in C16:0, C22:0 C24:1 and C24:0 ceramides (Fig. S5).

**Figure 6:**
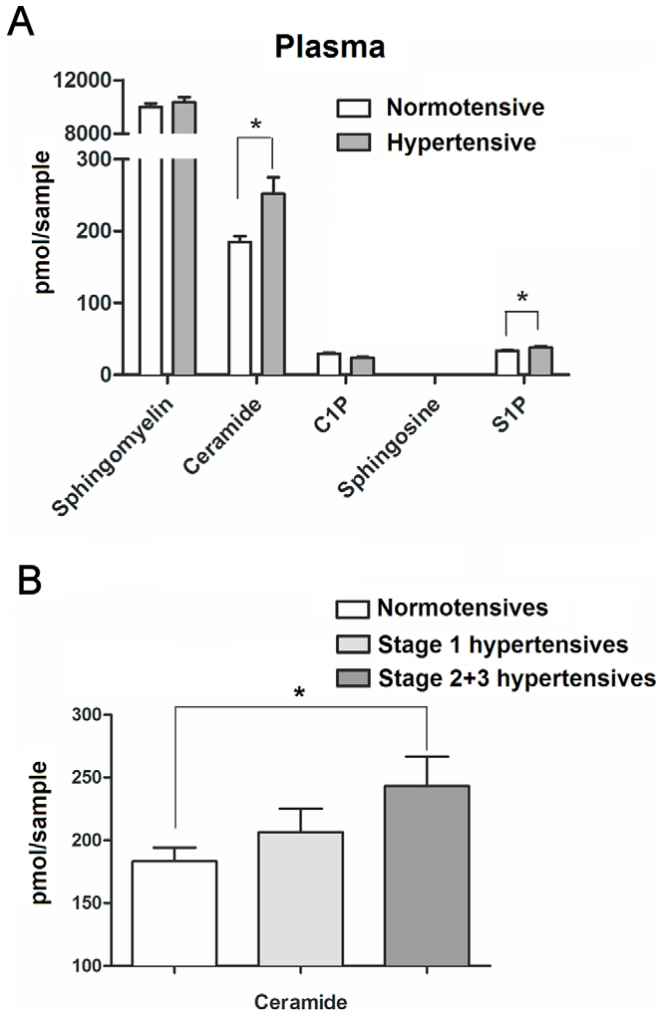
Liquid-chromatography-mass spectrometry measurements of sphingolipid content in human blood plasma. A) Quantification of total sphingolipid pools in human plasma. Human samples were collected from normotensive controls (BP<140/90 mmHg) and from patients with stage 2 and 3 hypertension (BP≥160/100). B) Total plasma ceramide levels in normotensives compared to stage 1 hypertensives (BP 140–159/90–99 mmHg) and stage 2+3 hypertensives. (Data presented as mean ± SEM, n = 18 for normotensive controls n = 12 for patients with stage 1 hypertension, n = 19 for stage 2+3 hypertension. (*) p<0.05.

In plasma of humans with stage 2+3 essential hypertension, ceramide levels were significantly higher compared to healthy normotensive controls (243.2±23.5 pmol vs 183.2±11.1 pmol respectively, n = 18–19, p<0.05; Fig. 6A). Moreover, ceramide levels correlated with increasing severity of hypertension, with ceramide levels in humans with stage 1 hypertension being intermediate of those from normotensives and stage 2–3 hypertensives (Fig. 6B). The distribution pattern of sphingolipids in plasma of humans was virtually identical of that found in rats. Increases in C24:1 and C24:0 ceramides mainly accounted for the observed increase in total plasma ceramide in hypertensive humans (Fig. S5). In contrast to significantly altered hypertensive patient plasma S1P levels compared to normotensive patients (37.0±1.8 pmol vs 32.0±1.2 pmol respectively, p<0.05), no significant changes in plasma S1P in rats were seen (355.6±17.1 pmol vs 312.1±16.7 pmol respectively, p>0.05). Whether this reflects species differences or differences in sample collection remains to be determined [13]. In respect of sphingomyelin and C1P levels, no significant changes where found in both human and rat samples with the given sample size, although a trend of decreased C1P plasma levels was observed in human hypertensives (29.0±1.7 pmol normotensives vs 26.0±1.9 pmol hypertensives, p>0.05).

## Discussion

Previous studies from our group and others have shown that sphingolipids are involved in the regulation of the release or action of endothelium-derived relaxing factors (NO and EDHF) [8], [14], [15]. Here we show for the first time that 1) Elevation of vascular ceramide leads to vasoconstriction due to increased TXA_2_ release in vessels of SHR. 2) These constrictions are only observed in vessels of SHR due to increased expression of enzymes involved in thromboxane A_2_ synthesis. 3) That basal ceramide levels are elevated in both SHR and humans with hypertension.

### Altered sphingolipid biology in hypertension

Because sphingolipids have vasoactive properties and play a pivotal role in cellular growth, we hypothesized that the sphingolipid system is involved in hypertension, a condition associated with altered vascular contractility and remodeling. Alterations in sphingolipid biology in hypertension were exemplified by the fact that pharmacological modulation of vascular sphingolipid composition, by means of the application of the sphingosine kinase inhibitors DMS or DHS, induced pronounced transient contractile responses in isolated carotid arteries from SHR but not from WKY. In analogy to sphingosine kinase inhibition, also the exogenous application of SMase induced contractions in carotid arteries from SHR, but only minor responses in arteries from WKY.

### Mechanism of SMase and DMS-induced contractions

Simultaneous application of DMS and SMase induced stronger arterial contractions than either component alone, but without signs of synergy, suggesting common pathways. Both modulators likely induce an accumulation of ceramide as a shared mechanism, as has been shown for SMase [16] and DMS in several cellular systems [17], [18]. Interestingly, the transient contractions induced by both DMS and SMase proved to be endothelium-dependent since mechanical removal of the endothelium abolished the contractions. In contrast, pre-incubation with the NO synthase inhibitor L-NAME augmented these contractions, indicating that the contractions are not due to a reduced NO bioavailability.

This endothelium dependency presents similarities to the “classical” endothelium-derived contractile factor (EDCF) described in the vasculature of SHR and human patients with essential hypertension [1], [19], [20]. Impaired relaxing responses to acetylcholine in both SHR and human essential hypertension involve the release of a COX-1-derived EDCF. The prostanoids PGH_2_, PGI_2_ and thromboxane A_2_ have been suggested as the EDCFs responsible for increased vascular tone in hypertension [2].

In the present study, both DMS- and SMase-induced contractions in SHR carotid arteries could be decreased by selective COX-1 inhibition but not by inhibition of COX-2. Furthermore, the fact that TXAS inhibition attenuated SMase-induced contractions, suggests that under our experimental conditions the contractions are caused by the generation of TXA_2_. Although the involvement of PGI_2_ is unlikely, its contribution cannot be fully excluded [21]. The prominent role of TXA_2_ is further supported by the observation that SMase-induced contractions were concentration-dependently inhibited by the TP-receptor antagonist SQ29548. The possibility that SHR thromboxane signaling is potentiated due to increased TP receptor expression or affinity in VSMCs is unlikely because the concentration-response-curves for the thromboxane analogue U46619 were comparable in SHR and WKY arteries. This is also in line with other studies, showing no TP receptor expression changes in SHR [22]. Thus, it seems that modulation of endothelial sphingolipid composition by ceramide elevation induces a COX-1-dependent release of TXA_2_ in vessels from SHR. Immunohistochemical analysis indicated elevated COX-1 expression in SHR vascular smooth muscle cells, which is supported by findings of Ge *et al.* [23], that smooth muscle cells from SHR aorta display elevated COX-1 mRNA expression. In addition to COX-1, we observed that also TXAS protein expression is increased in SHR, as has been previously shown by Tang *et al.* [22], at the mRNA level. The increased TXAS protein expression reached statistical significance in SHR carotid artery endothelium.

### The link between sphingolipid metabolism and eicosanoid synthesis

In the process of EDCF generation, substrate delivery to COX-1 depends primarily on PLA_2_ activity (for recent reviews see Vanhoutte *et al.* [24] and Feletou *et al.* [2]). Three PLA_2_ subtypes have been described thus far. While secretory PLA_2_ (sPLA_2_) and cytosolic PLA_2_ (cPLA_2_) require calcium for activation, calcium-independent PLA_2_ (iPLA_2_), located in both cytosolic and membrane fractions, does not require Ca^2+^ directly for catalytic activity [25]. Importantly, sphingolipids have previously been implicated in PLA_2_ activation; both ceramide and C1P have been shown to activate sPLA_2_ and/or cPLA_2_ *in vitro* [26], [27]. However, in the present study both single and combined addition of non-specific cPLA_2_ and sPLA_2_ inhibitors did not affect SMase-induced contractions. The iPLA_2_-specific inhibitor BEL nevertheless, was effective in this respect, suggesting that endogenous iPLA_2_ may contribute to the contractile phenotype of arteries in SHR. This is also in line with the recent findings of Wong *et al.* [28], and is further supported by our immunohistochemical finding that the endothelium in SHR (compared to WKY) expresses significantly more iPLA_2_, while levels appeared lower in the smooth muscle layer of the artery segments. This results in a remarkable increase in the ratio of endothelium/smooth muscle iPLA_2_ expression in the carotid arteries of SHR. That ceramides are able to activate iPLA_2_ is supported by findings of Gong *et al* [29]. In the present study imaging mass spectrometry and experiments with SMaseD revealed that ceramide (and not C1P) is most likely responsible for iPLA_2_ activation (Fig. S2, Fig. S3).

### Pathophysiological role of sphingolipids in hypertension-associated endothelial dysfunction

The aforementioned findings indicate that ceramide participates in the prominent role of thromboxane A_2_ in the SHR. Our lipidomics (LC-MS) analysis revealed that in arterial tissue of SHR ceramide levels were significantly higher when compared to normotensive WKY rats. It is tempting to speculate that the elevated basal arterial ceramide levels observed in our study contribute to endothelial dysfunction in SHR since these may lead to constitutive TXA_2_ production. In this regard, it is noteworthy that thromboxane receptor antagonism completely restored endothelial function in SHR. Furthermore, increased ceramide levels in hypertension may possibly be derived from elevated angiotensin II type 2 receptor signaling, which has been linked to ceramide production (for review see Berry *et al.* [30]) Results from another study by Johns *et al.* indicated decreased levels of ceramide in smooth muscle cells of SHR [31]. This discrepancy may be due to the fact that we determined ceramide levels directly in freshly isolated vessels whereas Johns *et al.* [31] used cultured smooth muscle cells between passages 3 and 12, which is known to induce phenotypic changes including changes in sphingolipid signaling [32].

The physiological relevance of ceramide-induced thromboxane A_2_ release (Fig. S6) is reflected by the observation that *in vivo* infusion of DMS resulted in increased arterial resistance and blood pressure in SHR, but not WKY rats. This marked blood pressure elevation was probably not due to cardiac effects of DMS since we observed a concomitant decrease in heart rate. The rise in systemic blood pressure indicates that, in addition to large conduit vessels such as the carotid artery, also resistance vessels of SHR are sensitive to DMS. Although the contractions to DMS in isolated carotid arteries were not due to inhibition of NO production, as indicated by the augmented contractile response in the presence of L-NAME, a possible inhibitory effect of DMS on NO production *in vivo* in other vascular beds cannot be excluded. The DMS-induced pressor response clearly emphasizes the importance of altered sphingolipid biology in vascular tone and blood pressure regulation *in vivo* in hypertensive rats.

Interestingly, the altered sphingolipid biology and elevated arterial ceramide levels in the vasculature of SHR are also reflected in increased plasma ceramide levels. Moreover, analysis of plasma from hypertensive- and normotensive humans revealed similar elevations in ceramide levels in patients with essential hypertension. Ceramide plasma levels showed a stepwise increase with increasing severity of hypertension, with ceramide levels in patients with stage 1 hypertension being intermediate of those from normotensives and stage 2–3 hypertensives. This implies that similar pathophysiological mechanisms in human hypertension may contribute to increased vascular tone and endothelial dysfunction. Of interest, very recently, a genetic analysis by Fenger *et al.* also suggested the involvement of the ceramide/S1P rheostat in the blood pressure regulation in human hypertension on a genetic basis [33].

In summary, we provide new insight in the pathophysiological role of sphingolipids in endothelial function and hypertension. We demonstrate that elevation of vascular ceramide in SHR induces a marked endothelium-dependent release of TXA_2_ that may contribute to endothelial dysfunction in hypertension. A prerequisite for this contractile response to ceramide is the increased arterial expression of enzymes involved in TXA_2_ synthesis as observed in vessels from hypertensive animals. Moreover, basal ceramide levels are increased in both SHR and humans with hypertension. The present study does not allow us to draw conclusions on causality. Since the development of hypertension in SHR precedes the development of endothelial dysfunction it is unlikely that the alterations in sphingolipid levels are the primary causative factor of hypertension. Nevertheless, both our *in vitro* and *in vivo* data clearly demonstrate that these alterations in sphingolipid biology can contribute to an increased vascular tone. Further research of the role of sphingolipids in the pathophysiology of human essential hypertension is therefore warranted.

## Materials and Methods

### Ethics statement

Written informed consent was obtained from all participants, and the study was approved by the local Research Ethics Committee of the Academic Medical Center.

The experiments involving animals in this study followed a protocol approved by the Animal Ethical Committee of the University of Amsterdam (DFC101766) and Maastricht University (2008-139), The Netherlands, in accordance with EU regulation on the care and use of laboratory animals.

### Human subjects

Blood plasma was obtained from otherwise healthy age-matched treatment-naïve patients with stage 1 hypertension (n = 12) or stage 2 and 3 hypertension (n = 19) and normotensive controls (n = 18). Patient characteristics are summarized in table 2. Blood pressure was measured three times following current guideline recommendations with an aneroid sphygmomanometer. The average of the last two blood pressure recordings was taken for analysis. Patients with confirmed or suspected of secondary hypertension, pregnant women and patients aged <18 years and patients with (a history of) alcohol abuse were excluded from participation.

**Table 2:** Patient characteristics.

Parameters	Normotensive	Stage 1 HT	Stage 2 and 3 HT
n	18	12	19
Age, years	44.1±2.5	44.1±2.8	47.4±2.6
MAP, mmHg	91.7±1.9	108.4±1.3 *	131.0±16.6 *#
Systolic BP, mmHg	121±2	143±3 *	171±6 *#
Diastolic BP, mmHg	77±2	91±1 *	111±3 *#
Male, n (%)	7 (39)	9 (75)	9 (47)
Black, n (%)	9 (50)	5 (42)	7 (37)
BMI, kg/m2	27.3±1.3	26.0±0.9	26.8±0.9
Diabetes, n (%)	0 (0)	0 (0)	1 (5)
Current smoking, n (%)	2 (11)	1 (8)	4 (21)

Mean arterial pressure (MAP), body mass index (BMI), blood pressure (BP). Data expressed as mean ±SEM or percentage of total (%), (*) p<0.05 vs normotensive subjects, and (#) p<0.05 vs Stage 1 hypertensives (HT).

### Animals

Adult six month old male Spontaneously Hypertensive rats (SHR) and Wistar Kyoto rats (WKY) were purchased from Charles River (Maastricht, The Netherlands). Rats were anaesthetized by i.p. injection of 75 mg/kg pentobarbital (O.B.G., Utrecht, The Netherlands). Heparin (750 IU, Leo Pharma B.V., Weesp, The Netherlands) was injected i.p. to prevent blood coagulation and thrombocyte-derived sphingosine-1-phosphate release. After tissue isolation, the animals were euthanized by exsanguination.

### Arterial preparation and isometric force recording

Carotid artery segments were isolated form 6 months old SHR and WKY rats and mounted into a wire myograph for isometric force measurements as described by Mulders *et al.* [9] (see Supporting Information S1). The sphingosine kinase inhibitor DMS (10 µmol/L) and the exogenous enzyme SMase (0.1 U/mL; from *Staphylococcus aureus*) or SMaseD (20 µL/OD_280_:0.4; from *Staphylococcus aureus*) were applied to segments to measure alterations in vasomotor tone within one hour. Inhibitors or antagonists were administered 30 min prior to these agents. In some experiments, the thromboxane/prostanoid receptor agonist U46619 was applied in half-log concentration increments.

### Immunohistochemistry

Enzyme expression in carotid artery segments of 6 months old WKY and SHR were quantified using a custom protocol developed in collaboration with Nikon Instruments Europe BV on unprocessed images (see Supporting Information S1).

### In vivo DMS administration

*In vivo* effects of DMS in isoflurane-anesthetized SHR and WKY were investigated by i.v. infusion (3 mg/kg, based on a pilot dose finding). Blood pressure, heart rate and carotid artery blood flow were recorded (see Supporting Information S1).

### Liquid chromatography - mass spectrometry of blood plasma and aorta

Blood plasma and aortic tissue from 6 months old SHR and WKY was isolated (Table 1). In addition, we assessed circulating sphingolipids in blood plasma obtained from otherwise healthy age-matched treatment naïve patients with stage 1 hypertension (n = 12) or stage 2 and 3 hypertension (n = 19) and normotensive controls (n = 18). Baseline characteristics according to blood pressure category are depicted in table 2. All samples were processed according to an established protocol as published by Merrill *et al.* [34] and Wijesinghe *et al.* [12] (see Supporting Information S1).

### Statistical data analysis

The isometric tension measurements in carotid artery segments are presented as mean ±SEM with ‘n’ being the number of individual rats. Peak contraction values (relative tension, mN/mm) during the experiments were gathered and expressed in column graphs. Column statistics were performed by one-way ANOVA including Dunnett's multiple comparisons test (95% confidence interval) with DMS or SMase values as control. The SMase controls were the same group of data for all appropriate figures. For protein quantification by IHC and lipid content quantification by LC-MS, Student's *t*-test was performed to compare single conditions between SHR and WKY or normotensive versus hypertensive subjects. Data measured *in vivo* were expressed as relative percentage and compared using one-way ANOVA including Tukey's multiple comparison test. All statistical analyses were performed using Prism (GraphPad Prism Software, San Diego, CA, USA). Values of p<0.05 were considered to be statistically significant.

## Supporting Information

Figure S1 **Concentration-response curve of the thromboxane analogue U46619 in SHR and WKY carotid artery.** Data presented as mean ± SEM, n = 4–6.
(TIF)

Figure S2 **Sphingomyelinase D-induced contractions in SHR carotid artery.** Quantification of SMaseD-induced contractions in SHR carotid artery, which was lower in WKY and comparable to SMaseC. Data presented as mean ± SEM, n = 2–4.
(TIF)

Figure S3 **Mass spectrometry imaging of lipids involved in SMase-induced contraction in SHR carotid artery.** A) High resolution total ion count image of SHR carotid artery sample (left, bottom scale bar 100 µm) and image of increased mass counts corresponding to treatment with SMase (depiction of SMase-treated segment total ion count minus untreated count; right image; blue) showing highest changes in luminal side of blood vessel (endothelial area). B) Discriminant analysis of untreated and SMase-treated tissue categories: spectra are grouped per tissue and both tissue categories are separated along the discriminant function. Projection of mass spectra of standards (dots) on discriminant function (standards plotted above DA zero correspond with elevated presence after SMase treatment). C) Plot of the loadings for each mass channel in the direction of main separation between tissue groups (i.e. first DA function), showing masses (deviating from zero) that were elevated (top) or decreased (bottom) after SMase treatment. Sphingomyelinase C (SMase), non-treated (NT), ceramide-1- phosphate (C1P), sphingosine-1-phosphate (S1P).
(TIF)

Figure S4 ***In vivo*** **effects of DMS infusion in SHR and WKY.** Rats were treated with bolus injection and subsequent infusion of DMS (3 mg/kg followed by 6 mg/kg/hr) or vehicle (0.75% rat serum albumin in saline) during recording of A) carotid artery systolic blood flow (Peak flow) and B) heart rate (HR). Data expressed as mean maximal change from baseline ± SEM, n = 6–8, (*) p<0.05.
(TIF)

Figure S5 **Ceramide subspecies in human and rat tissue.** A) Plasma spectrum of measured ceramide subspecies in rat (SHR vs WKY) and normotensive vs. hypertensive patients. B) Rat aorta homogenate spectrum of ceramide subspecies. n = 6–19, (*) p<0.05.
(TIF)

Figure S6 **Potential mechanism of sphingolipid-mediated release of thromboxane A_2_ in SHR carotid artery.** Accumulation of ceramide by the sphingolipid modulators SMase and DMS induces thromboxane A_2_ production in an iPLA_2_, COX-1 and TXAS-mediated pathway. Upregulated enzyme expression or lipid levels in SHR carotid arteries indicated by white arrows.
(TIF)

Supporting Information S1 (DOC)
