# Supplementary figures and images for: Hypertension Is Associated with Marked Alterations in Sphingolipid Biology: A Potential Role for Ceramide
Source: PLoS One. 2011 Jul 19;6(7):e21817. doi: 10.1371/journal.pone.0021817 (PMC3139577; doi:10.1371/journal.pone.0021817)

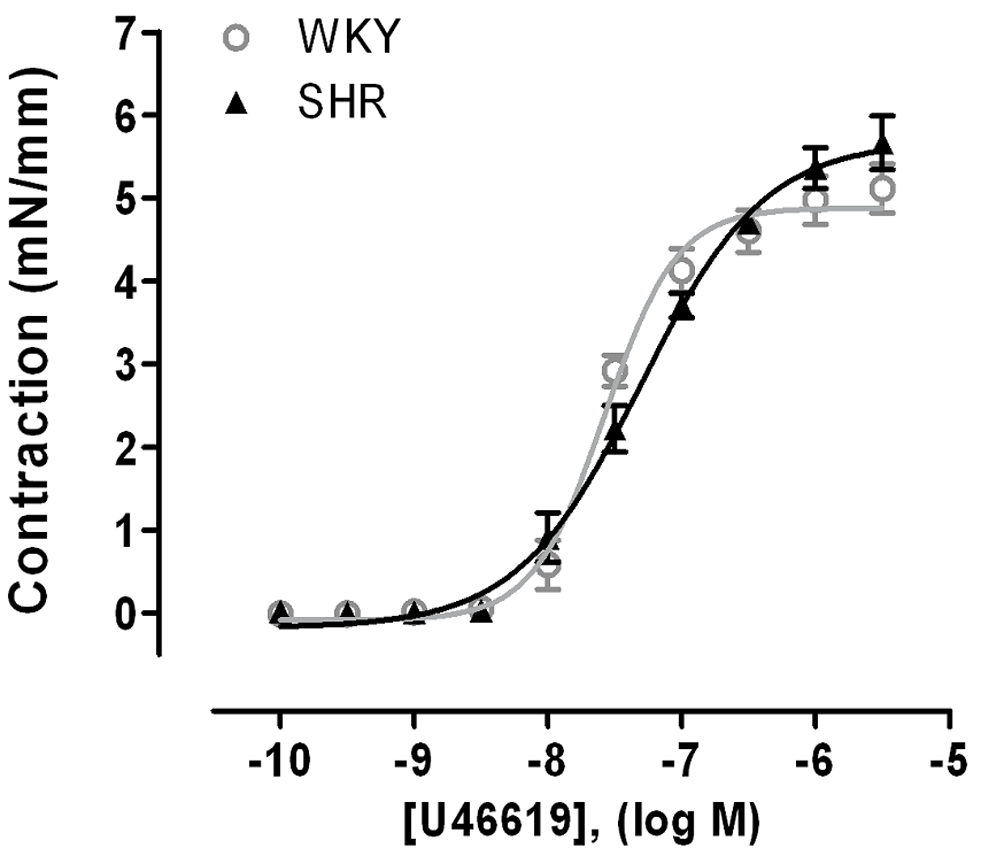

Supplement: Figure S1 — Concentration-response curve of the thromboxane analogue U46619 in SHR and WKY carotid artery. Data presented as mean ± SEM, n = 4–6. (TIF) [file pone.0021817.s001.tif]

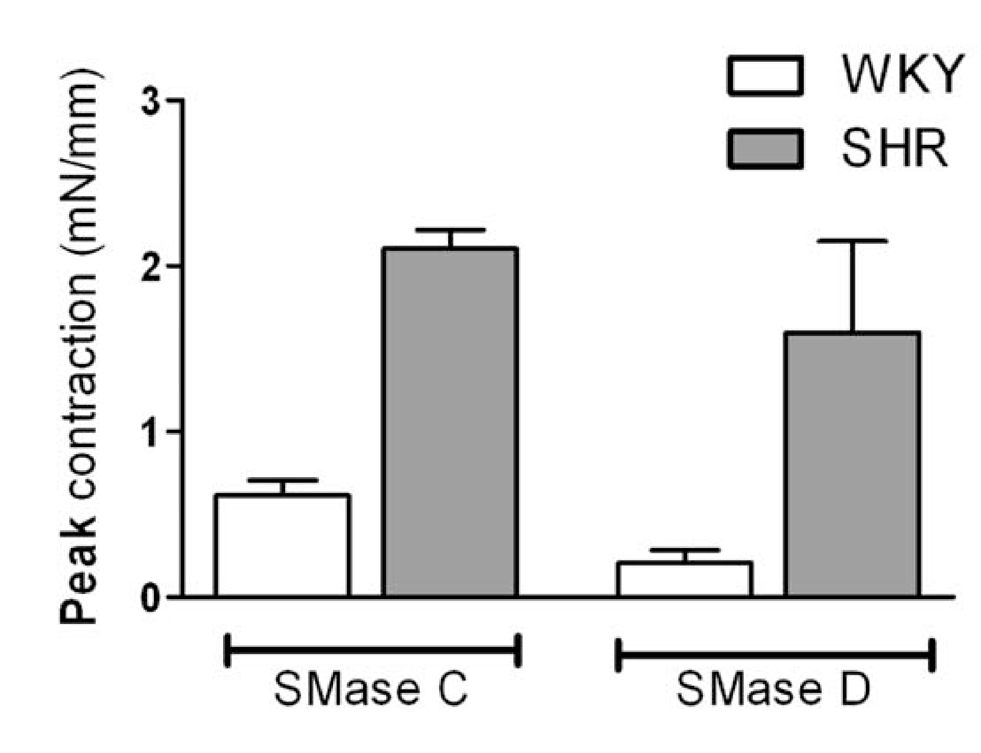

Supplement: Figure S2 — Sphingomyelinase D-induced contractions in SHR carotid artery. Quantification of SMaseD-induced contractions in SHR carotid artery, which was lower in WKY and comparable to SMaseC. Data presented as mean ± SEM, n = 2–4. (TIF) [file pone.0021817.s002.tif]

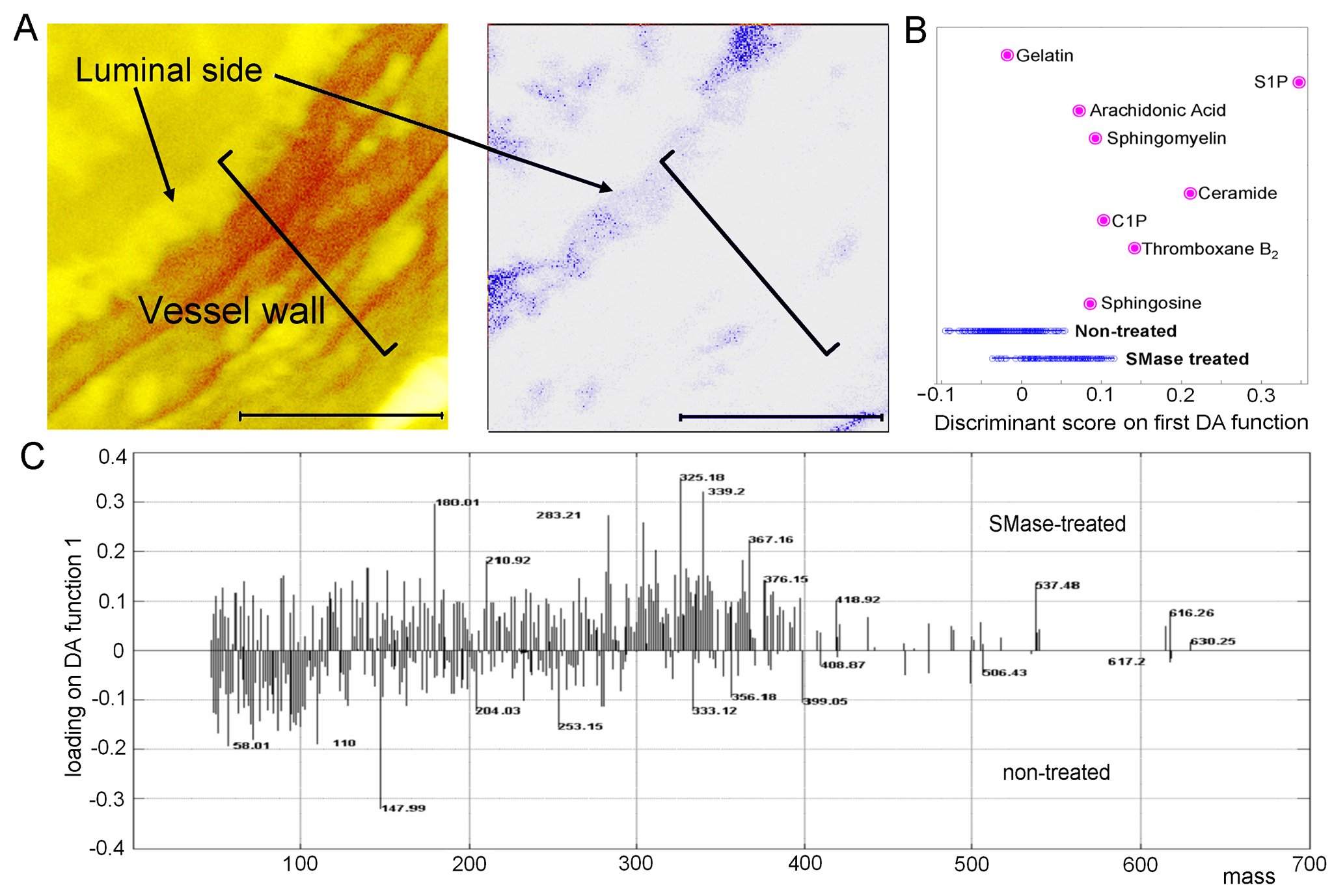

Supplement: Figure S3 — Mass spectrometry imaging of lipids involved in SMase-induced contraction in SHR carotid artery. A) High resolution total ion count image of SHR carotid artery sample (left, bottom scale bar 100 µm) and image of increased mass counts corresponding to treatment with SMase (depiction of SMase-treated segment total ion count minus untreated count; right image; blue) showing highest changes in luminal side of blood vessel (endothelial area). B) Discriminant analysis of untreated and SMase-treated tissue categories: spectra are grouped per tissue and both tissue categories are separated along the discriminant function. Projection of mass spectra of standards (dots) on discriminant function (standards plotted above DA zero correspond with elevated presence after SMase treatment). C) Plot of the loadings for each mass channel in the direction of main separation between tissue groups (i.e. first DA function), showing masses (deviating from zero) that were elevated (top) or decreased (bottom) after SMase treatment. Sphingomyelinase C (SMase), non-treated (NT), ceramide-1- phosphate (C1P), sphingosine-1-phosphate (S1P). (TIF) [file pone.0021817.s003.tif]

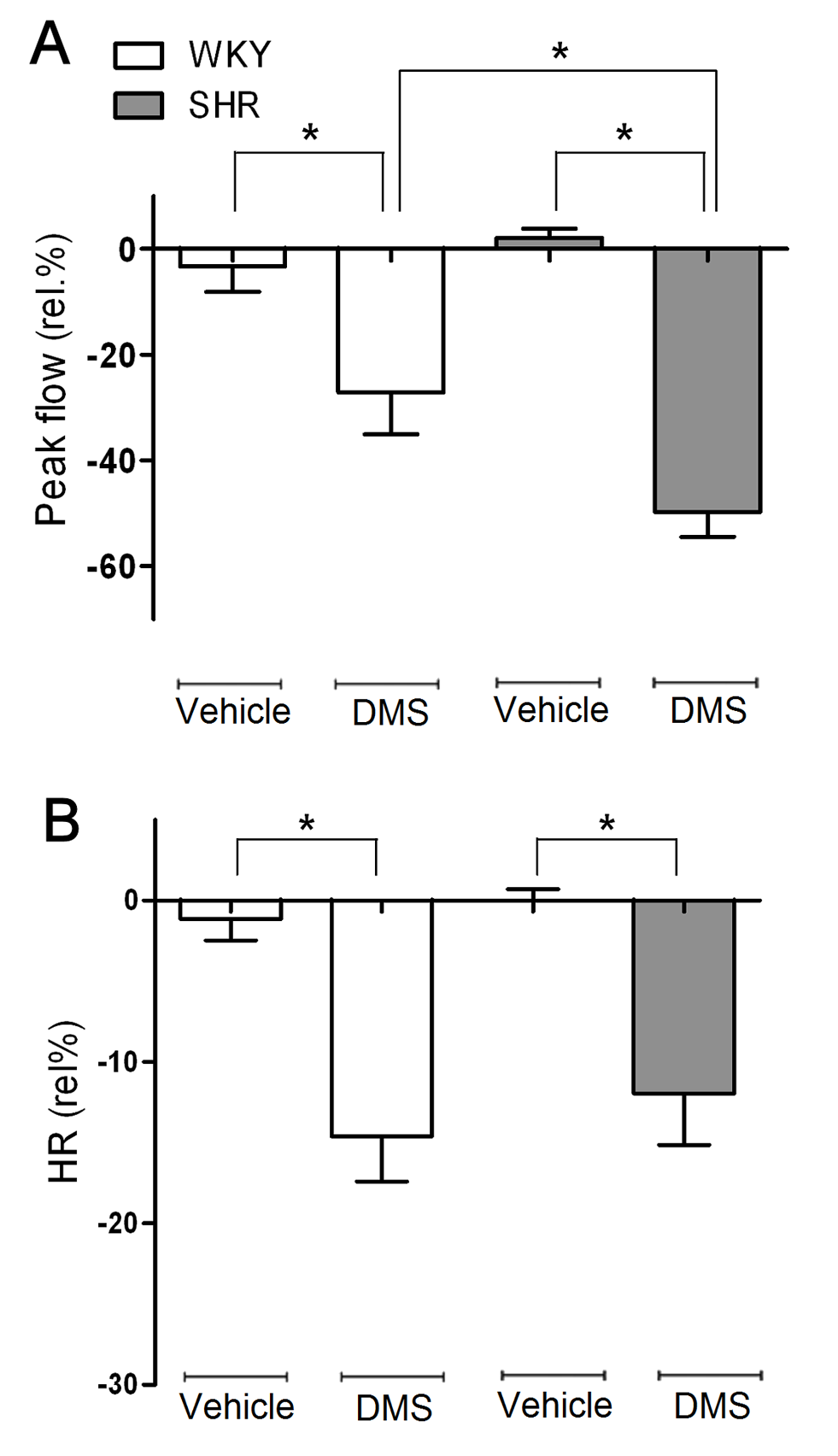

Supplement: Figure S4 — In vivo effects of DMS infusion in SHR and WKY. Rats were treated with bolus injection and subsequent infusion of DMS (3 mg/kg followed by 6 mg/kg/hr) or vehicle (0.75% rat serum albumin in saline) during recording of A) carotid artery systolic blood flow (Peak flow) and B) heart rate (HR). Data expressed as mean maximal change from baseline ± SEM, n = 6–8, (*) p<0.05. (TIF) [file pone.0021817.s004.tif]

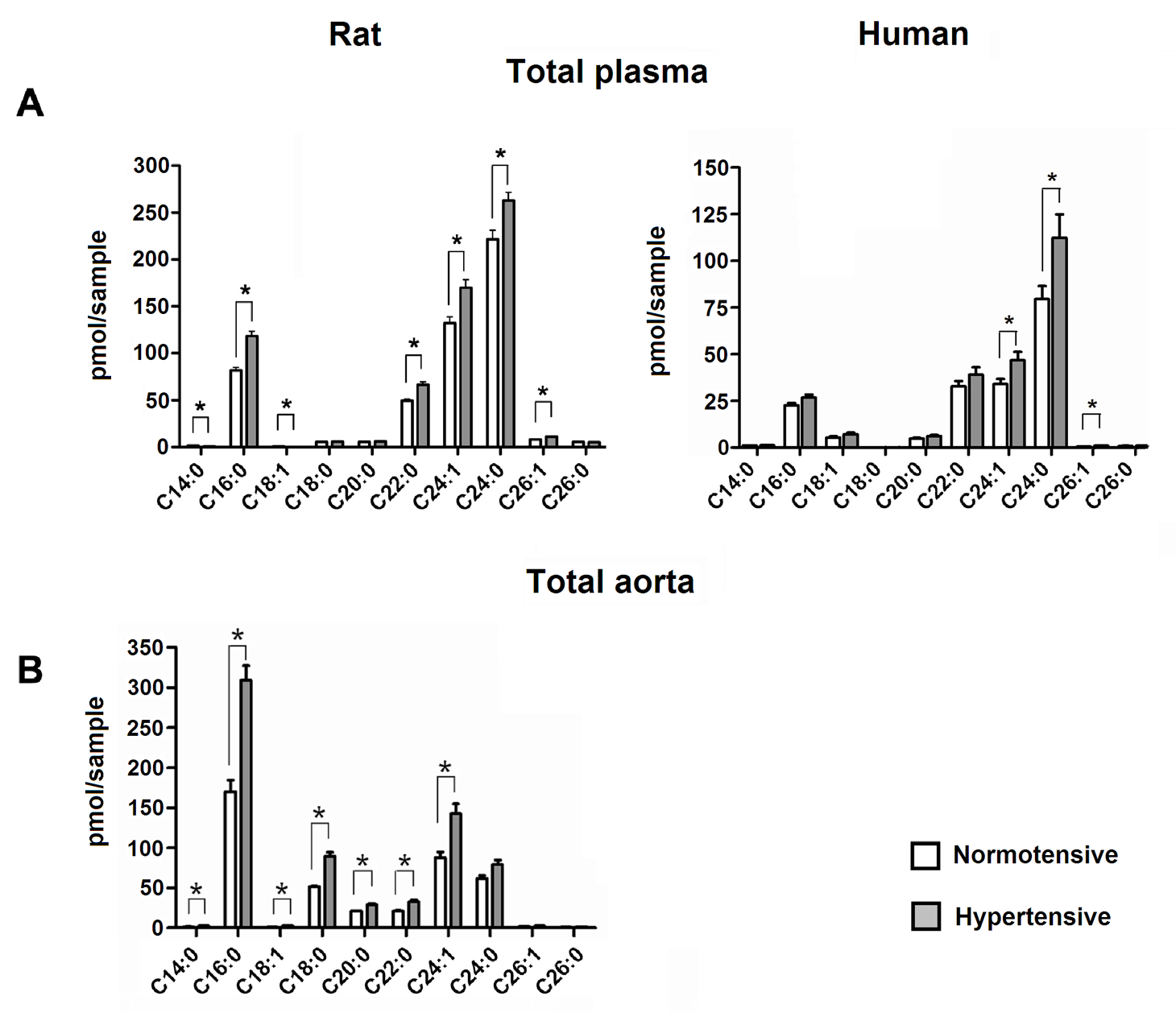

Supplement: Figure S5 — Ceramide subspecies in human and rat tissue. A) Plasma spectrum of measured ceramide subspecies in rat (SHR vs WKY) and normotensive vs. hypertensive patients. B) Rat aorta homogenate spectrum of ceramide subspecies. n = 6–19, (*) p<0.05. (TIF) [file pone.0021817.s005.tif]

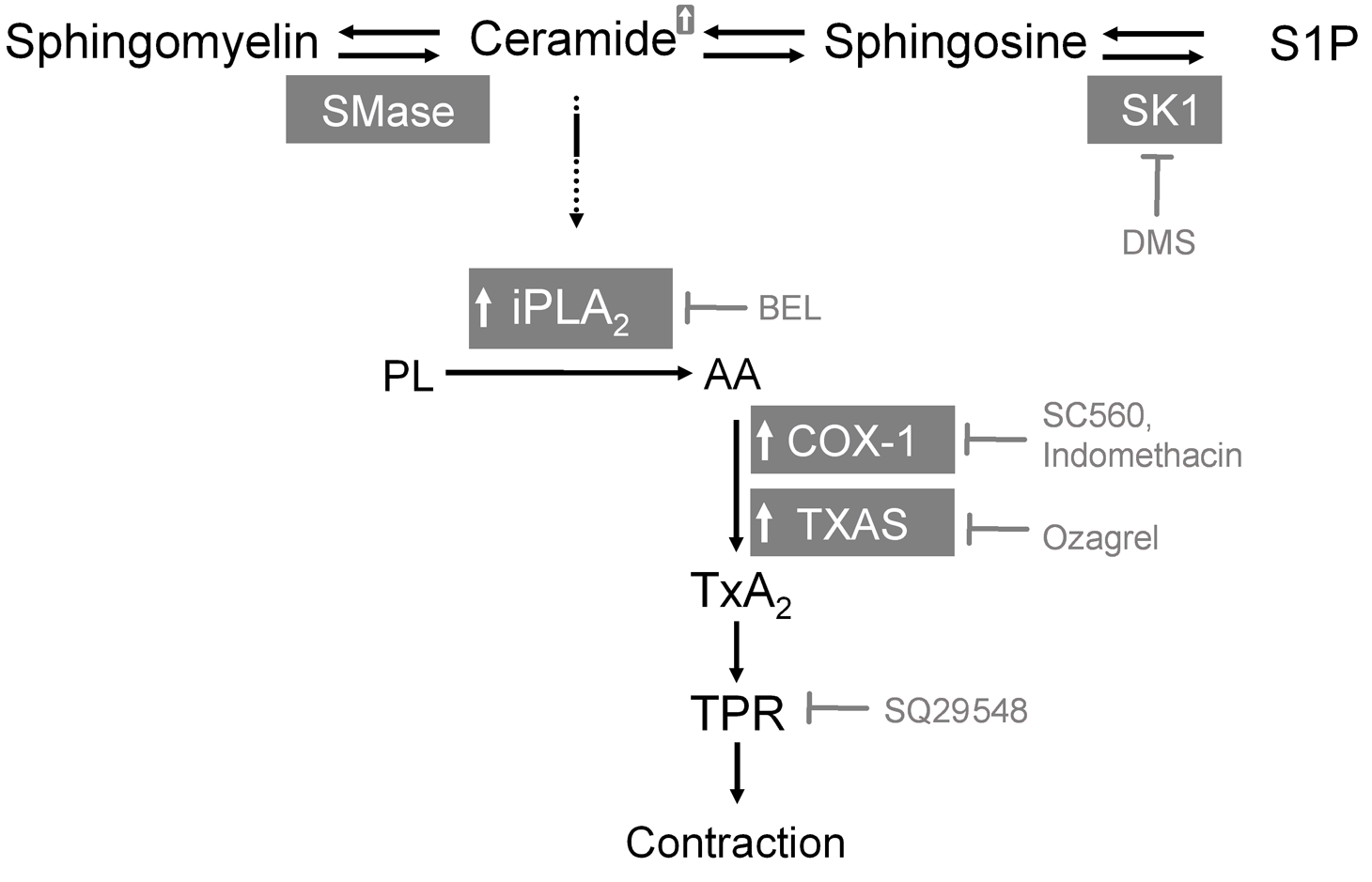

Supplement: Figure S6 — Potential mechanism of sphingolipid-mediated release of thromboxane A2 in SHR carotid artery. Accumulation of ceramide by the sphingolipid modulators SMase and DMS induces thromboxane A2 production in an iPLA2, COX-1 and TXAS-mediated pathway. Upregulated enzyme expression or lipid levels in SHR carotid arteries indicated by white arrows. (TIF) [file pone.0021817.s006.tif]
