# Supplementary material for: Hypertension Is Associated with Marked Alterations in Sphingolipid Biology: A Potential Role for Ceramide
Source: PLoS One. 2011 Jul 19;6(7):e21817. doi: 10.1371/journal.pone.0021817 (PMC3139577; doi:10.1371/journal.pone.0021817)
Supplement: Supporting Information S1 — (DOC) [file pone.0021817.s007.doc]

**Information S1**

*Compounds and antibodies*

Acetyl-β-methylcholine (methacholine; MCh), phenylephrine (Phe), indomethacin (Indo; COX inhibitor), Nω-Nitro-L-arginine methyl ester (L-NAME; NOS inhibitor), Ozagrel (TXAS inhibitor) and Bromoenol lactone (BEL; iPLA_2_ inhibitor) were purchased from Sigma-Aldrich Chemical Co. (St. Louis, MO, USA). D-erythro-N,N-dimethylspingosine (DMS; SK inhibitor), DL- threo-dihydrosphingosine (DHS; SK inhibitor), neutral sphingomyelinase C (SMase; from *Staphylococcus aureus*) and arachidonyl-trifluoromethyl ketone (AACOF3; cPLA_2_ inhibitor) from Biomol International L.P. (Plymouth, PA, USA). SC-560 (COX-1 inhibitor), thromboxane B2, arachidonic acid and U46619 (thromboxane analogue) from Cayman Chemical Co. (Ann Arbor, MI, USA). Luffariellolide (Luff; sPLA_2_ inhibitor) and SQ29548 (SQ29; TP receptor antagonist) from Alexis Biochemical (San Diego, CA, USA). NS-398 (COX-2 inhibitor) from Tocris Bioscience (Bristol, UK). Sphingomyelinase D (SMaseD, optical density OD280: 0.4; from *Staphylococcus aureus*) was a kind gift of Prof. Zhe Lu, Department of Physiology, University of Pennsylvania, USA. Sphingomyelin d18:1/18:0, sphingosine d18:1, sphingosine-1-phosphate d18:1, ceramide d18:1/18:0, ceramide-1-phosphate d18:1/18:0 for mass spectrometry were purchased from Avanti Polar Lipids (Alabaster, AL, USA).

Antibodies against cyclooxygenase 1 (order#160109; 1/400 dilution used) and thromboxane synthase (#160715; 1/200) were purchased from Cayman Chemical. Ceramide kinase (ab38011; 1/50) antibodies from Abcam (Cambridge, UK). Calcium-independent phospholipase A2 (LS- B1603; 1/200) antibody from LifeSpan Biosciences (Seattle, WA, USA). Von Willebrand factor (A0082; 1/250 and GTX74830; 1/200) antibody from DacoCytomation (Glostrup, Denmark) and GeneTex (Irvin, CA, USA) respectively. Alexa Fluor 488 (A-11029) and (A-11055), Fluor 546 (A-11010) and (A-21085) antibodies (all 1/400) from Invitrogen (Carlsbad, CA, USA).

*Liquid chromatography - mass spectrometry of blood plasma and aorta*

Post-anesthesia, the thoracic region was opened and blood was collected by cardiac puncture using a 21G needle (BD Microlance 3) and a pre-chilled (0°C) polypropylene blood collection tube, filled with 200 µL of PECT solution containing: prostaglandin E1 (94 nmol/L), Na2CO3 (0.63 mmol/L), EDTA (90 mmol/L) and theophylline (10 mmol/L). Blood samples (2 mL) were collected in these tubes via an open system, drop by drop to avoid platelet activation *ex vivo* and immediately placed on ice. Blood plasma was prepared by centrifugation for 20 min at 1600 x g, 4°C within 10 min after collection and stored at -80°C until further processing. Furthermore, the abdominal aorta was isolated and snap-frozen in liquid nitrogen. Then, two aortas were pooled for every sample, grinded in liquid nitrogen using a mortar, and dissolved in 700 µL PBS.

For blood plasma samples, lipids were extracted from 33 µL (rat) or 95 µL (human) plasma as described by Wijesinghe *et al.* [1] and Merrill *et al.* [2] with slight modifications. Briefly; to 200 µL of plasma 1 mL methanol and 0.5 mL chloroform were added together with an internal standard containing 500 pmol of the following; d17:1 sphingosine, sphinganine, sphingosine-

1-phosphate and sphinganine-1-phosphate, and d18:1/12:0 ceramide, ceramide-1-phosphate, sphingomyelin and glucosylceramide. The mixture was sonicated and incubated at 48°C overnight. The following day, extracts were subjected to base hydrolysis for 2 hrs at 37°C using 150 µL of 1 mol/L methanolic KOH. Following base hydrolysis the extract was completely neutralized by the addition of glacial acetic acid. The neutralization was confirmed by pH measurement. Half of the extract was dried down and brought up in reversed phase sample buffer (60%A:40%B) . To the remainder of the extract 1mL chloroform and 2mL water were added, and the lower phase was transferred to another tube, dried down and brought up in normal phase sample buffer (98%A:2%B). Sphingosine, sphinganine, sphingosine-1-phosphate sphinganine-1-phosphate and ceramide-1-phosphate were quantified via reversed phase HPLC ESI-MS/MS using a Discovery C18 column attached to a Shimadzu HPLC (20AD series) and subjected to mass spectrometric analysis using a 4000 Q-Trap (Applied Biosystems) as described by Wijesinghe *et al*. (2009). Ceramides, sphingomyelins and monohexosyl ceramides were quantified via normal phase HPLC ESI-MS/MS using an amino column (Sigma) as described by Merrill, Jr. *et al*. (2005). For aorta samples, lipids were extracted from 500 µL of a 10% solution of the tissue in PBS according to Wijesinghe *et al.* [1] and Merrill *et al.* [2] with slight modifications. Briefly to 500 µL of the 10% tissue homogenate 2 mL of methanol and 1 mL of chloroform was added together with an internal standard and processed as described above. Finally, the quantified lipids were normalized to the volume of the material injected into the column.

*Arterial preparation and isometric force measurement*

Rat common carotid arteries were excised distal from their bifurcation from the aortic arch and placed in carbogen (95% O2, 5% CO2) aerated Krebs-Henseleit buffer (pH7.4; 118.5 mmol/L NaCl, 4.7 mmol/L KCl, 25.0 mmol/L NaHCO3, 1.2 mmol/L MgSO4, 1.8 mmol/L CaCl2, 1.1 mmol/L KH2PO4 and 5.6 mmol/L glucose) at room temperature. After removing connective and adipose tissue, vessels were cut into segments of 2mm in length and two stainless steel wires (40 µm in diameter; Goodfellow Huntington, U.K.) were inserted intralumenally to mount in a multi channel wire myograph organ bath (M610, Danish Myo Technology A/S, Aarhus, Denmark) containing pre-warmed (37°C) Krebs-Henseleit buffer under continuous carbogen aeration for isometric force measurement. For endothelium- denuded vessel measurements, polyethylene tubing (PE-10; Clay Adams) was inserted after segment cutting and rolling force was applied five times. After equilibration of the vessels during 20 min, arterial lumen diameters were normalised according to Mulvany & Halpern (1977) and as previously described [3]. During normalisation, all segments were individually stretched until the internal circumference was 90% of which the segments would have at transmural pressure (100mmHg). Then, vessel segments equilibrated during 30 min before starting with a training protocol. During the entire protocol, organ bath buffer was replaced every 15 min (Krebs, 37°C, aerated) when applicable. Vessels were exposed to high K+ containing Krebs buffer (pH 7.4; 23.2 mmol/L NaCl, 100 mmol/L KCl, 25 mmol/L NaHCO3, 1.2 mmol/L MgSO4, 1.8 mmol/L CaCl2, 1.1 mmol/L KH2PO4 and 5.6 mmol/L glucose), evoking contraction that was allowed to stabilize during 15 min. Vessels were rinsed with Krebs buffer to gain baseline tension during 30 min and high K^+^ contraction was repeated with subsequent wash out. Then, vessels were pre-contracted with the α1-adrenoceptor agonist phenylephrine (0.5 - 1 µmol/L) inducing a contraction averaging 60 - 80% of that induced by high K^+^. Relaxation was induced by adding methacholine (10 µmol/L) which gave an indication of endothelial function or denudation efficiency. Subsequently, after incubation with fresh Krebs buffer during 30 min, a third high K^+^ contraction was induced.

*Immunohistochemistry and quantification*

Rat carotid artery segments were collected directly after dissection. Segments were rapidly submerged in OCT Compound (Sakura, TissueTek) and frozen in liquid nitrogen with subsequent storage at -80°C. Frozen sections (5 µm thick) were cut on a Leica CM3050S cryostat and dried by cold pressurized air before storage at -80°C. Upon defrosting tin-foil wrapped sections, slides were fixed in 100% acetone during 15 min. Then, slides were washed shortly in 0.1% PBS/BSA (w/v) and incubated with blocking buffer (2% PBS/BSA or 5% PBS/serum of appropriate 2^nd^ antibody) during 30 min at RT. After a short wash, slides were incubated with the primary antibody dissolved in 0.1% PBS/BSA overnight at 4°C. Following a triple wash in 0.1% PBS/BSA during 5 min, the appropriate secondary antibody was applied during 1 hour at RT. After triple wash, the antibody against von Willebrand Factor (vWF) was applied during 1 hr at RT as marker of the endothelium. After triple wash, the final fluorescent antibody was applied. Finally after triple wash, DAPI containing mounting medium (UltraCruz, sc-24941) was applied and vessels were imaged at room temperature using a Nikon Eclipse TE2000-U fluorescence microscope (Plan Fluor ELWD 20x objective, Nikon DXM1200F digital camera) with NIS Elements AR 2.30 software. During imaging, the region of interest was located in each vessel by proper detection of the endothelial marker vWF, without any information on the protein to quantify to ensure unbiased recording. Then the appropriate filter setting was chosen to record the accompanying protein intensity. Quantification of fluorescence was performed using the NIS Elements software in agreement with a tailor-made Nikon protocol on the raw unprocessed images. Briefly, using the endothelial marker, a region was selected and copied over the protein to quantify the intensity, yielding a mean intensity of fluorescence for endothelial cells. Then, the tunica media was selected and mean fluorescence intensity was determined for smooth muscle cells. For both determinations, an intensity threshold was selected to exclude background fluorescence. All settings and exposure times were applied to all slides equally for the appropriate protein to quantify. Figure 3 depicted images were processed after quantification on raw data by Corel Paint Shop Pro X v10.3.

*Time-of-flight imaging secondary-ion mass spectrometry*

Vessel segments from the myograph organ bath after pharmacological stimulation or no stimulation were frozen in 10% gelatin in liquid N2, transported on dry ice and stored at -80°C upon usage. Importantly, SMase-treated vessel segments were frozen upon reaching the peak of contraction, ensuring maximal detection of involved lipids. Just before use, arteries were transferred to a cryomicrotome were they were allowed to warm up to -20°C. Then, 10 µm thick sections of the arteries were prepared and immediately thaw-mounted on indium-tin- oxyde (ITO) conductive glass slides. Sections were allowed to warm up to room temperature and dry by placing the ITO slides for 10 min in a dessicator. Without further treatment, slides were then directly mounted in a sample holder and immediately inserted into the mass spectrometer. All procedures were undertaken with gloves and alcohol/hexane-cleaned tools in order to avoid any contamination of the sample. Two sets of sample types were analyzed: a non-treated SHR artery (4 repeats) and a SHR artery treated with SMase (2 repeats). Repeats (images acquired independently) were done on several sections from one artery per sample type.

Standards of sphingosine, sphingosine-1-phosphate, ceramide, ceramide-1-phosphate, sphingomyelin, arachidonic acid and thromboxane were used. Then, 0.5 µL droplets of standard solutions were spotted on different substrates: ITO glass, steel, and gelatin and allowed to dry before analysis by SIMS. Static SIMS imaging experiments were performed in a Physical Electronics (Eden Prairie, MN) TRIFT-II TOF SIMS instrument equipped with a gold liquid metal ion gun. All experiments were performed with 22 kV Au+ primary ions providing on stage a current of around 500 pA with ion pulse length of 18 ns. Secondary ions were extracted by a 3.5 kV extraction voltage from the ion source into the TOF analyzer and post accelerated with an additional 7kV prior to detection on a dual multichannel plate detector.

Images were obtained by randomly rastering during 1200 seconds, the focused primary ion beam across a 200x200 µm2 area chosen on the artery wall. Standard spectra were obtained by imaging standard droplets in a pattern of 8x8tiles of 200x200 µm2 per tile during 2 seconds per tile. Lipids investigated forming preferentially negative ions, the 0-1000 mass-to-charge ratio (*m/z*) range was recorded in negative ion mode. Mass spectra were calibrated using low mass fragment ions: H^-^, C^-^,CH^-^, CH2^-^, CH3-, O^-^, OH^-^, Cl^-^, PO3^-^ H2PO4^-^, and checked on near ubiquitous fatty acid chain C16H31O2-. Raw images were recorded with a spatial resolution of 256×256 pixels, with an actual spatial resolution of about 1 µm on tissue. Because of the high mass resolution of the SIMS data, the number of mass channels was reduced to enhance stability of the signal and speed up the PCA and DA calculations. Peak picking and integration was performed using the PEAPI algorithm as described by Eijkel *et al.* (2009) [4]. Each image dataset was first converted from RAW image format into a single mass spectrum with 0.01 *m/z* bin size. All spectra were combined in a single file in order to perform a single peak picking step on all spectra and obtain a single common peak list. It was important for further analysis that all images were described within the same spectral data space, i.e. with the same peak list. Then each RAW image was filtered with the common peak list and converted into a 64x64 pixel image (8x8 for standards).

Principal component analysis (PCA) and Discriminant analysis (DA) were performed using the in-house built ChemomeTricks toolbox for MATLAB version 7.0 (The MathWorks, Natick, MA, USA). Principal Component Analysis (PCA) enabled the selection of tissue areas from

200 µm-side field of view images of the artery wall. Here, we only considered MS data that correlated to tissue areas from the ion image (i.e. MS data from the embedding medium around tissue was selectively discarded). PCA is a widely used multivariate data analysis method, described in numerous articles [5,6]. PCA reduces the dimensionality of the dataset by the creation of a new set of variables, the principal components (PCs). These PCs are linear combinations of the original variables, in this case mass channels from mass spectra. Correlated variables (i.e. mass channels originating from the same chemical compound) were grouped into the same PC. The PCs were hierarchically sorted by the amount of variance they describe. The first PC (PC1) explained the largest amount of the variance; noise-related signals, describing low amounts of variance, were found in the higher-ranked PCs. Discarding these higher-ranked PCs from further data processing greatly reduced the noise in the data. The number of relevant PCs was determined by summation of the variances described per PC until 80% of the total variance was accounted for. The remaining, higher ranked, PCs were discarded from further analysis.

Discriminant analysis (DA) was performed on the resulting PCs using the double stage principal component analysis as described by Hoogerbrugge *et al.* [7]. Since the spectra in the dataset were picked from distinct groups (tissue types, treatment conditions) they were assigned to separate groups. DA used the group information to enhance the separation between these groups by maximizing the between-groups variance and minimizing the within-group variance. This resulted in Discriminant Functions (DFs) that were linear combinations of PCs. The DFs were hierarchically sorted by the between-within variance ratio (B/W).

*In vivo DMS administration*

SHR and WKY rats were anaesthetized with isoflurane (2.5–4 v/v%) during the entire experiment. At the end of the study the animals were euthanized with an intravenous bolus pentobarbital (200 mg/kg). Blood pressure was measured intra-arterially via a canula inserted into the abdominal aorta via the left femoral artery. DMS was dissolved via sonication in rat serum albumin (RSA)-enriched saline. Two PE canula's were inserted into the femoral vein. One *iv.* line was used for the continuous infusion of DMS (0.5%DMS/0.75%RSA/saline) or its vehicle solution (0.75% RSA/saline). The left common carotid artery was exposed and connected to a TranSonic Transit time flow probe (Transonic Systems Europe, Maastricht, NL). Both arterial pressure and carotid arterial blood volume flow (mL/min) were recorded a 2.5 kHz using IDEEQ data acquisition software (Maastricht, The Netherlands) and stored on hard disk for further analysis. After reaching a steady baseline on both flow and pressure recordings, vehicle or DMS were infused in bolus (3 mg/kg DMS) as determined by a pilot dose-finding study of 0.3-1-3-10 mg/kg DMS. This dose was unlikely to be toxic since in a study by Shirahama *et al.*, mice were treated several days with a higher dose of DMS without any sign of toxicity [8]. Peak effects of DMS on blood flow and arterial pressure, observed between 5-10 min, were selected.

**Supplemental results**

Both, ceramide and C1P are known activators of secretory and/or cytosolic PLA2 . In order to investigate whether ceramide or C1P (generated via phosphorylation of ceramide by ceramide kinase) is responsible for the iPLA2-dependent contraction we made use of the special properties of SMaseD, a sphingomyelinase isoform that can be found in certain spider species and bacteria. In contrast to SMase, SMaseD generates C1P directly from sphingomyelin by releasing the choline head group. Also SMaseD induced strong contractions in arteries from SHR and to a much lesser extend in those of WKY (Fig. S2). The onset of contraction was, however, strongly delayed compared to SMase (SMaseC: ~10 min versus SMaseD: >30 min.) and more variable in amplitude. SMaseD-induced contraction was, like SMase-induced contractions, sensitive to inhibition by BEL.

Secondary Ion Mass Spectrometry (SIMS)-imaging measurements were performed on SHR carotid artery slices to confirm a possible role of ceramide or ceramide-1-phosphate in SMase-induced contractions. Tissue features could be identified from high spatial resolution total ion images (Fig. S3A left), and SMase-induced changes are mainly found in the endothelial area (Fig S3A right). The separation between SMase-treated and non-treated tissue groups suggested that there were significant spectral (hence chemical) differences between those two tissue groups (Fig. S3B+C). From all standards, especially S1P and ceramide had high scores and others, among which C1P, appeared less relevant for tissue group separation (Fig. S3B). This suggests that ceramide is more likely to be involved in SMase-induced contraction in carotid arteries from SHR than is C1P. Taken together, in addition to altered sphingolipid levels, the increased arterial expression of enzymes involved in the EDCF pathway is a prerequisite for arterial contraction in response to ceramide (Fig. S6).

**Supplemental references**

1. Wijesinghe DS, Allegood JC, Gentile LB, Fox TE, Kester M, et al. (2009) Use of high pressure liquid chromatography, electrospray ionization-tandem mass spectrometry for the analysis of ceramide-1-phosphate levels. J Lipid Res 51: 641-651.

2. Merrill AH, Jr., Sullards MC, Allegood JC, Kelly S, Wang E (2005) Sphingolipidomics: high-throughput, structure-specific, and quantitative analysis of sphingolipids by liquid chromatography tandem mass spectrometry. Methods 36: 207-224.

3. Mulders AC, Hendriks-Balk MC, Mathy MJ, Michel MC, Alewijnse AE, et al. (2006) Sphingosine kinase-dependent activation of endothelial nitric oxide synthase by angiotensin II. Arterioscler Thromb Vasc Biol 26: 2043-2048.

4. Eijkel GB, Kaletas BK, van der Wiel IM, Luider TM, Heeren RMA (2009) Correlating MALDI and SIMS imaging mass spectrometric datasets of biological tissue surfaces. Surface and Interface Analysis 41: 675-685.

5. Chou Y-L (1969) Statistical Analysis, with Business and Economic Applications. 17.9 New York: Holt, Rinehart And Winston, Inc.

6. Wall ME, Rechtsteiner A, Rocha LM (2003) Singular value decomposition and principal component analysis. In: Berrar DP, Dubitzky W, Granzow M, editors. A practical approach to microarray data analysis. Norwell, MA: Kluwer Academic Publishers. pp. 91-109.

7. Hoogerbrugge R, Willig SJ, Kistemaker PG (1983) Discriminant analysis by double stage principal component analysis. Anal Chem 55: 1710-1712.

8. Shirahama T, Sweeney EA, Sakakura C, Singhal AK, Nishiyama K, et al. (1997) In vitro and in vivo induction of apoptosis by sphingosine and N, N-dimethylsphingosine in human epidermoid carcinoma KB-3-1 and its multidrug-resistant cells. Clin Cancer Res 3: 257-64.
